# Quantum Thermalization Dynamics of Fermi Gases Quenched to the BEC‐BCS Crossover

**DOI:** 10.1002/advs.202507343

**Published:** 2025-05-29

**Authors:** Licheng Yi, Shuxian Yu, Meimei Wu, Shujin Deng, Haibin Wu

**Affiliations:** ^1^ State Key Laboratory of Precision Spectroscopy, Insitute of Quantum Science and Precision Measurement East China Normal University Shanghai 200062 P. R. China; ^2^ Shanghai Branch Hefei National Laboratory Shanghai 201315 China; ^3^ Collaborative Innovation Center of Extreme Optics Shanxi University Taiyuan 030006 China; ^4^ Shanghai Research Center for Quantum Sciences Shanghai 201315 China

**Keywords:** quench dynamics, quantum thermalization dynamics, universality

## Abstract

Understanding nonequilibrium dynamics of strongly interacting quantum systems represents one of the most challenging problems in many‐body physics. Here, quantum thermalization dynamics are explored in real‐time in an ultracold Fermi gas suddenly quenched to the BEC‐BCS crossover. When quenched to unitarity, it is observed that the cloud size remains unchanged in the early evolution while the momentum distribution emerges two prethermal states with a lifetime difference of two orders of magnitude in the early and intermediate stage before very slowly evolving to the final stationary state. It is revealed that a crossover momentum, at which the momentum distribution remains nearly unchanged, is determined by the thermal wavelength at high temperatures and the Fermi momentum while at low temperatures. It is identified that the universal prethermal dynamics scaling where momentum distributions with different temperatures collapse onto one curve. When quenched to the BEC side, the thermalization rapidly relaxes into a prethermal state and exhibits the low energy oscillation related to the molecular bound states. This work provides benchmarks for the study of quantum thermalization in strongly interacting fermionic many‐body systems.

## Graphical abstract

This study, reporting the first experimental realization of interaction quench far faster than the Fermi timescale in fermionic many‐body systems, explores real‐time quantum thermalization dynamics in the BEC‐BCS crossover. It observes prethermal states with distinct lifetimes after quantum quench, uncovers universal prethermal scaling, and observes rapid relaxation on the BEC side, advancing many‐body physics benchmarks.

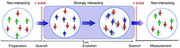

## 1 Introduction

The nonequilibrium dynamics of the strongly interacting fermionic systems is closely related to the neutron stars, high‐temperature superconductivity and quark‐gluon plasma. Understanding these complex dynamics represents one of the unsolved problems in the modern many‐body physics.^[^ ^1^, ^2^, ^3^ ^]^ Ultracold Fermi gas provides an ideal platform to explore such dynamics owing to its unprecedented controllability and long coherent time. For the out of equilibrium dynamics, the Fermi energy *E*_*F*_ sets the shortest time of the collective collisional response of the fermionic many‐body systems through a Fermi time τ_*F*_ = ℏ/*E*_*F*_, where ℏ is a reduced Planck constant. A paradigmatic protocol in nonequilibrium dynamics is quantum quench, in which a Hamiltonian parameter is suddenly changed. Although it is conceptually simple, there is generally difficult to understand that how strongly interacting systems relax to equilibrium.^[^ ^1^, ^2^ ^]^

Although the isolated quantum system seems impossible to completely thermalize after the quench due to the unitary time evolution, it has been recognized that the system would quickly relax to a long‐lived non‐thermal state, known as a prethermalized state.^[^ ^4^ ^]^ This phenomenon has been observed in many quantum systems.^[^ ^5^, ^6^, ^7^, ^8^, ^9^, ^10^, ^11^ ^]^ Recently, the quenching dynamics has been observed in bosonic atomic systems, exhibiting universal scaling.^[^ ^12^, ^13^, ^14^, ^15^ ^]^ However, little is known about the subsequent evolution of the system after trapped in such quasi‐stationary states, due to the formidable technical and theoretical challenge that poses the nonequilibrium dynamics of many‐body systems. For example, the large three‐body collisions and recombination heating in the strongly interacting bosonic atoms hinder the further understanding of such complex thermalization in the long timescales.

On the other hand, the strongly interacting Fermi system is collisionally stable and the three‐body collision is greatly suppressed.^[^ ^16^ ^]^ The Fermi condensation, pairs momentum and Higgs amplitude mode have been experimentally investigated by an interaction modulation or a magnetic field quench at a time scale of tens of τ_*F*_.^[^ ^17^, ^18^, ^19^ ^]^ The dynamics of quasiparticles of impurities coupled to an atomic Fermi sea is observed by using fast interferometry.^[^ ^20^ ^]^ The quenching dynamics over a superradiant quantum phase transition with a Fermi gas in the cavity has been investigated.^[^ ^21^ ^]^ Recently, the prethermalization and energy dynamics are predicted by an interaction quench faster than τ_*F*_,^[^ ^22^, ^23^, ^24^ ^]^ showing a controllable suppression in the Fermi momentum discontinuity near the Fermi surface and oscillation of the two‐body contact. In spite of numerous theoretical and experimental efforts to study the out of equilibrium dynamics of strongly interacting Fermi gases, a direct experimental observation of the relaxation dynamics with the interaction quench faster than τ_*F*_ is still elusive.

Here we observe the out of equilibrium dynamics of an ultracold Fermi gas quenched from the non‐interacting regime to the BEC‐BCS crossover in a timescale faster than τ_*F*_. The two‐photon Raman coupling technique enables us to quickly prepare, quench and detect the system between the non‐interacting and strongly interacting Fermi gas. The post‐quench thermalization dynamics is explored in real time by measuring the momentum and spatial distribution, which shows very complex evolution stages with different timescales. Two prethermalized states emerge with very different lifetimes in the early and intermediate stages when the Fermi gas is quenched to unitarity. In the long time, the momentum distribution relaxes very slowly. There exists a characteristic momentum at which the momentum distribution is almost not evolved. We demonstrate the universal prethermal dynamics with a new scaling for different temperatures. When quenched to the BEC side, the momentum distribution shows an oscillation behavior, whose frequencies are determined by the energy of the bound states. We also reveal that the cloud size exhibits different dynamics in real space, which is not changed when the momentum experiences a violent relaxation in the early stage.[Supplementary-material advs70188-supl-0001]

## 2 Results and Discussion

**Figure 1:**
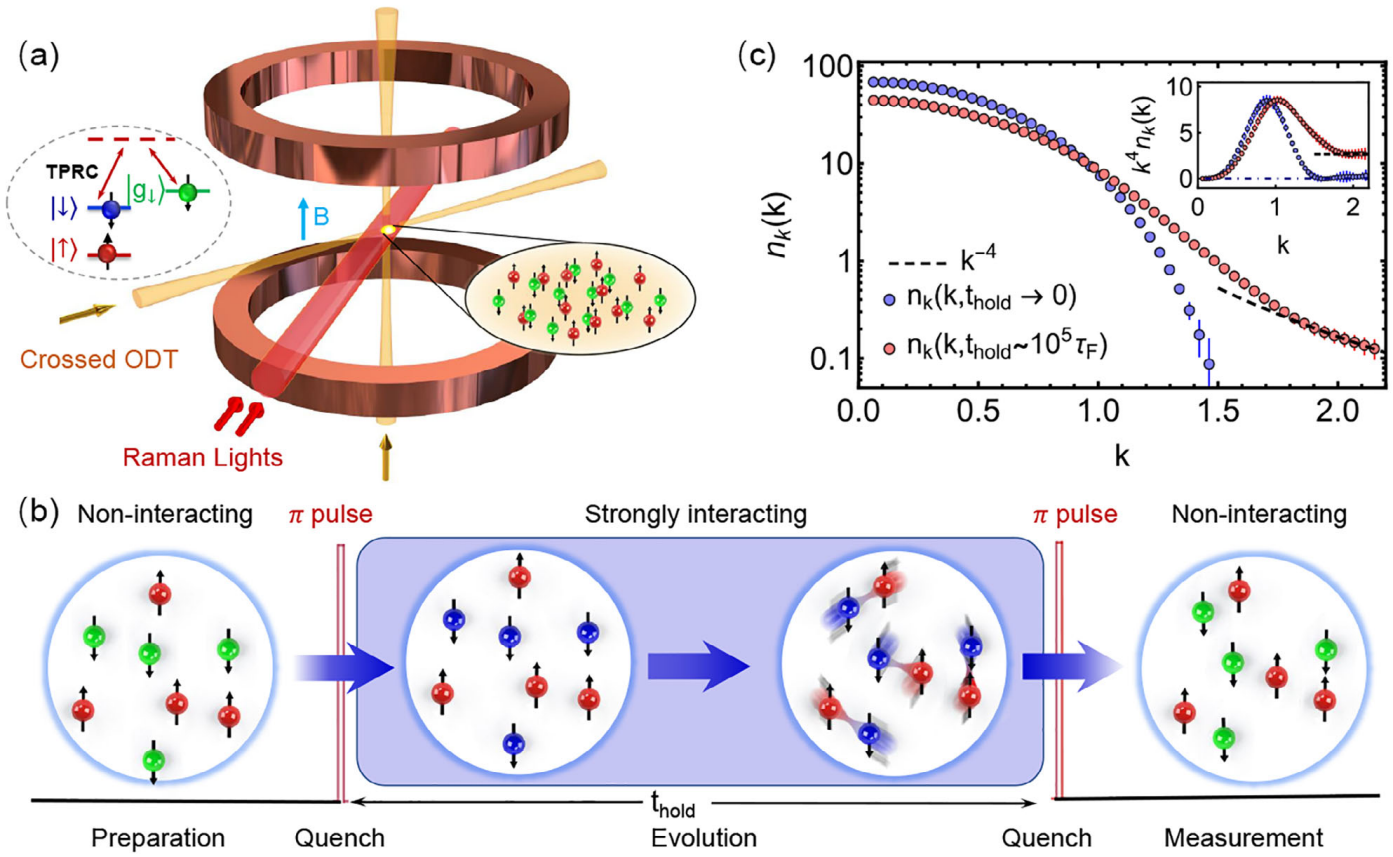
The experiment procedure and typical momentum distribution for the post‐quench dynamics. a) The schematic of the experimental setup. The interaction of atoms in |↑〉 and |↓〉 states is tuned by the Feshbach resonance with a magnetic field *B*. The two‐photon Raman coupling (TPRC) between |↓〉 and |*g*_↓_〉 state quickly realizes the interaction quench faster than τ_*F*_. ODT is the optical dipole trap. b) The experimental procedure. The Fermi gas is initially prepared in the non‐interacting regime. Then a Raman π pulse is used to suddenly quench the gas into unitary, BEC and BCS regime. Subsequently, after evolving for a holdtime *t*_*hold*_, the gas is quenched back into the non‐interacting regime again with another Raman π pulse. The absorption images are performed to extract the momentum and density distribution. c) The typical momentum distributions for different *t*_*hold*_, which show the dynamical evolution for different *k* states and a emergent *k* ^−4^ scaling (dashed line) for the large momenta with the long *t*_*hold*_. The inset shows *k* ^4^ *n*_*k*_(*k*) for *t*_*hold*_ = 0 and *t*_*hold*_ = 10^5^τ_*F*_, where the dotdashed line represents *k* ^4^ *n*_*k*_(*k*) = 0.

For the experiment, we employ an ultracold Fermi gas of ^6^Li in a harmonic trap. The schematic is shown in **Figure** **1**a. A balanced mixture of ^6^Li fermions is initially prepared in the two lowest hyperfine states |↑〉 and |↓〉, which is loaded into a crossed dipole trap to evaporatively cool to quantum degeneracy, similar to our previous work.^[^ ^25^, ^26^ ^]^ Then, by a two‐photon Raman π pulse, atoms in the state |↓〉 are completely transferred into another hyperfine state |*g*_↓_〉 with opposite electronic spin and the same nuclear spin, for which the interaction with the residual atoms |↑〉 is negligible. After the gas is held 2s into the equilibrium, an ideal Fermi gas with an effective Fermi energy $E_{F}\equiv \hbar \bar{\omega }{\left(6N\right)}^{1/3}$ is prepared, where $\bar{\omega }$ is the geometric mean of the trap frequencies, *N* is the atom number per spin, respectively. The corresponding Fermi time τ_*F*_ = 4.63 µ*s* sets the natural time scale. Combined the magnetically Feshbach resonance and the second Raman π pulse to transfer the atoms from |*g*_↓_〉 to |↓〉 state, we quench the Fermi gas from the non‐interacting regime to the BEC‐BCS crossover with a quenching time *t*_*q*_ = 330 *ns* ≪ τ_*F*_ and then let it evolve for a time *t*_*hold*_.^[^ ^27^ ^]^ After different evolving time *t*_*hold*_, we quench the Fermi gas back to the non‐interacting regime with another π pulse, release it from the harmonic trap and measure its momentum distribution by an absorption image for a sufficiently long time of flight (*t*_*tof*_) expansion. Each experiment is repeated for 40–200 times with the same conditions to suppress the fluctuations. The atom images are performed by the radially averaged inverse Abel transformation to obtain the momentum distribution *n*_*k*_(*k*), which is normalized by ∫*n*_*k*_(*k*)/(2π)^3^ *d* ^3^ *k* = 0.5, where *k* is the wavenumber in units of the Fermi wavenumber $k_{F}=\sqrt{2mE_{F}}/\hbar$, *m* is the atomic mass.

We first present the experimental observation for the universal thermalization dynamics of the Fermi gas quenched to the unitarity in the momentum space. After the quench, the population experiences quite different dynamics with different momentum states, as shown in Figure 1c. The atoms for the small momenta keep decreasing while those for the large momenta show an opposite increasing trend in the evolution. *n*_*k*_(t) clearly reveals a *k* ^−4^ tail in large momenta for *t*_*hold*_ > 10τ_*F*_, which is closely related to the contact in the strong interaction regime.^[^ ^28^ ^]^ This observation is in contrast to the case of the BEC where the asymptotic form *n*_*k*_(t) ∼ *k* ^−4^ is not observed at very high *k*.^[^ ^12^, ^15^ ^]^

**Figure 2:**
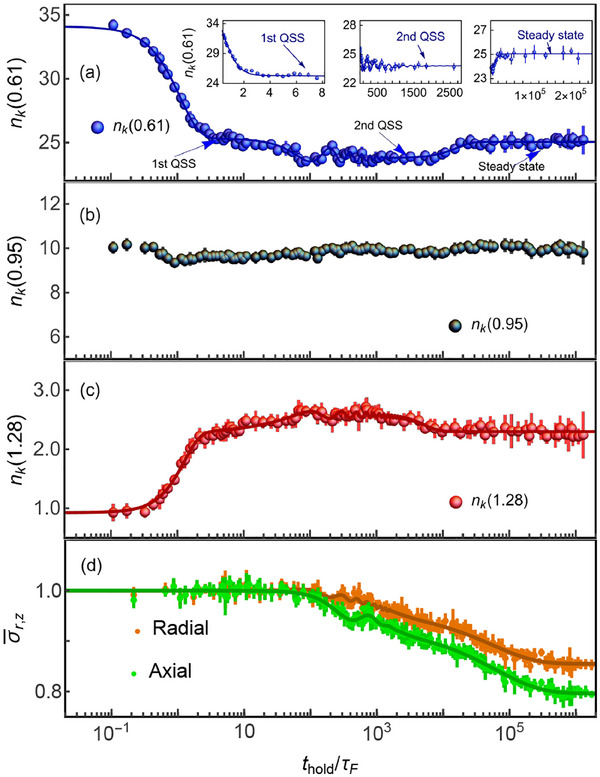
Momentum distribution with different *k* states and the mean cloud size for Fermi gases quenched to unitarity. The dynamical evolution of *n*_*k*_(0.61) (a) and *n*_*k*_(1.28) (c), which show a quick initial decrease and increase, then reaches a 1st QSS, subsequently experiences a damped oscillation with a collective mode. After this oscillation, they enter the 2nd QSS with a long lifetime of about thousands of the Fermi time τ_*F*_. Subsequently, *n*_*k*_ slowly thermalizes towards its final stationary state on a much longer timescale. Insets are the closer inspections for 1st QSS, 2nd QSS, and steady states, respectively. b) The evolution of *n*_*k*_(0.95), which shows nearly unchanged. d) The dynamics of the mean cloud size. Dots are measured data and solid lines are corresponding fitting curves with a sigmoid function.^[^ ^27^ ^]^

By investigating individuals *k*, the evolution of *n*_*k*_ exhibits different stages with different timescales, as presented in **Figure** **2**. Two prethermalized states emerged in the early and intermediate dynamical evolution, respectively. The post quench dynamics lasts a very long time evolution to reach its final steady state, about 2 × 10^4^τ_*F*_ (0.1 *s*).^[^ ^27^ ^]^ The instantaneous momentum distribution *n*_*k*_ changes very fast within several τ_*F*_. In this stage, two‐body collisions dominate the dynamics, *n*_*k*_ experiences a rapid decreasing for small momentum (Figure 2a) and growth at the large momentum (Figure 2c). After this violent relaxation, *n*_*k*_ reaches a plateau, a quasi‐stationary state (QSS). After remaining in this state about tens of τ_*F*_, *n*_*k*_ begins the evolution and experiences a damped oscillation with a collective mode (about 1.83 times the radial trap frequency), where the Fermi gas is the collisional and viscous hydrodynamics. When the oscillation is damped out, *n*_*k*_ stays on a long‐lifetime prethermalized state (the second QSS) with about thousands of τ_*F*_. Subsequently, *n*_*k*_ slowly thermalizes towards its final stationary state on a much longer timescale, about the magnitude of 10^4^ τ_*F*_.

In the relaxation, the density distribution shows different dynamics with the momentum distribution. Being different from measuring its momentum distribution. We directly expand the atomic cloud to obtain the density distributions after the interaction quench to unitarity. In the early evolution of the system, although *n*_*k*_ experiences a violent relaxation, the density distributions remain nearly unchanged. The normalized mean cloud size ${\bar{\sigma }}_{r,z}=\sigma {\left(t_{hold}\right)}_{r,z}/\sigma {\left(0\right)}_{r,z}$ are displayed in Figure 2d, which clearly shows that ${\bar{\sigma }}_{r,z}$ does not change in the range of *t*_*hold*_/τ_*F*_ = 0 ∼ 100, where σ(*t*_*hold*_)_*r*, *z*_ = 〈σ(*t*_*hold*_)_*r*, *z*_〉/*N* ^1/6^ is the atom number independent radial and axial mean cloud size, respectively. After this stage, ${\bar{\sigma }}_{r,z}$ exhibits the damped oscillation and enters into the QSS. Ultimately, ${\bar{\sigma }}_{r,z}$ very slowly relaxes to the values of the final steady state with a long characteristic time comparable with one in the momentum space.

Importantly, by closely checking *n*_*k*_, there is one special *k* at which *n*_*k*_ exhibits hardly any change in the evolution. *n*_*k*_ at *k* ≈ 1 (*n*_*k*_(0.95) is shown in Figure 2b) almost kept on a constant during the evolution, which is expected to show the Fermi momentum discontinuity at the prethermalization regime.^[^ ^22^ ^]^ No atom loss is observed until the evolution time reaches to about 10^5^ τ_*F*_.

**Figure 3:**
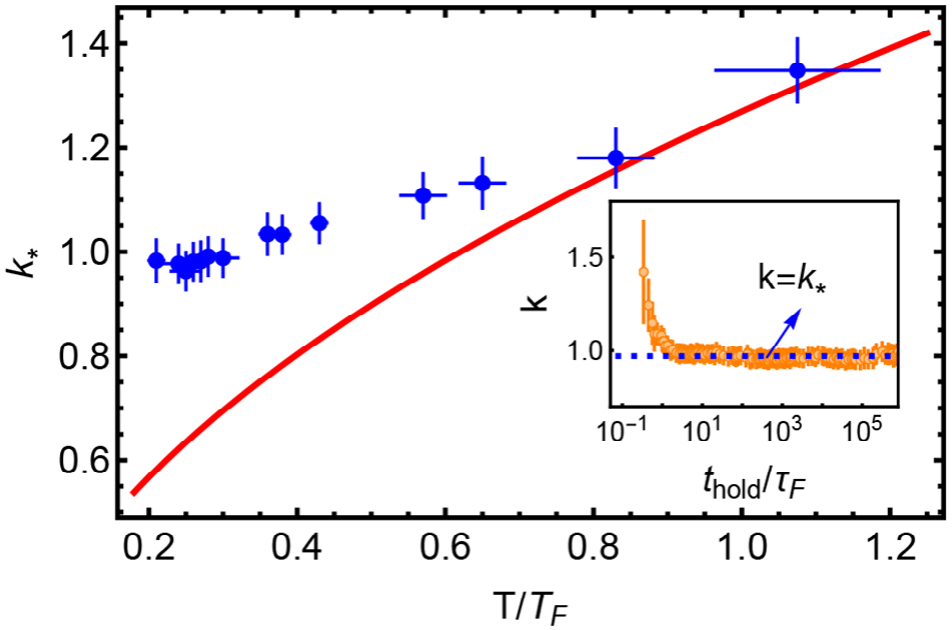
The measured k_*_ with different *T*/*T*_*F*_ in the unitary regime. Blue dots are data and solid red line is the theoretical prediction for a thermal bosonic quantum gas.^[^ ^29^ ^]^ The error bars represent the standard deviation of measurements. Inset is δ*n*(*k*) ≡ 0 as a function of *t*_*hold*_/τ_*F*_ and *k*. Dashed lines denote *k* = k_*_.

In order to further distinguish the crossover momentum, we investigate δ*n*(*k*) = *n*_*k*_(*k*, *t*_*hold*_) − *n*_*k*_(*k*, 0) ≡ 0 as the time evolves (the inset in **Figure** **3**). After several τ_*F*_, this crossover momentum approaches to an approximately fixed value k_*_.^[^ ^27^ ^]^ The dependence of the measured k_*_ on the dimensionless initial temperature *T*/*T*_*F*_ from 0.21 to 1.1 is shown in Figure 3, where *T*_*F*_ = *E*_*F*_/*k*_*B*_ is the Fermi temperature and *k*_*B*_ is the Boltzmann constant, respectively. The measured k_*_ in the dynamics of Fermi gases quenched to the unitarity exhibits different behavior with one for the bosonic atoms. For the BEC that atoms are condensed to the lowest or several low momentum states, the post‐quenching dynamics causes the redistribution of the population to higher momentum states and there is no explicit crossover momentum k_*_. For a thermal bosonic gas, k_*_ is proportional to the inverse of the thermal wavelength.^[^ ^12^, ^29^ ^]^ However, *k*_*F*_ naturally appears as another length scale for Fermi gases. In the high temperature, k_*_ is determined by the thermal wavelength, which agrees well with the perturbation theory (solid red curve), similar to the case of the thermal bosonic gas. Nonetheless, as the temperature is decreased, k_*_ deviates gradually from this behavior. In the low temperature, k_*_ approaches one (slightly smaller than 1 in Figure 3), indicating that momentum distribution near *k*_*F*_ remains unchanged and quantum thermalization redistributes the atoms with the momentum smaller than *k*_*F*_ to the momentum larger than *k*_*F*_.

**Figure 4:**
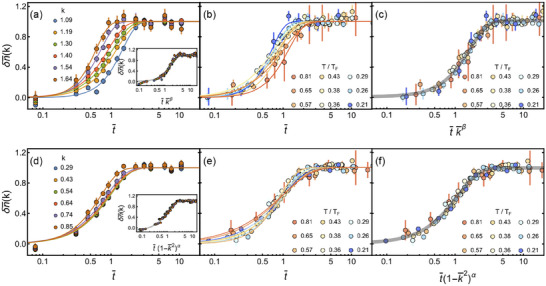
Universal scaling of the prethermal dynamics for Fermi gases quenched to unitarity with different reduced temperatures *T*/*T*_*F*_. (a) and (d) are the normalized momentum distribution difference $\delta \bar{n}\left(k\right)\equiv \delta n_{k}\left(t\right)/\bar{n}$ with the rescaled time $\bar{t}=t_{hold}/\tau_{F}$ at *T*/*T*_*F*_ = 0.26 for *k* > k_*_ and *k* < k_*_, respectively. Insets in (a) and (d) show $\delta \bar{n}\left(k\right)$ is collapsed into a single curve. (b) and (e) are $\delta \bar{n}\left(k\right)$ with $\bar{t}$ for difference *T*/*T*_*F*_ for *k* > k_*_ and *k* < k_*_, respectively. (c) and (f) are the universal scaling for $\delta \bar{n}\left(k\right)$ as a function of the rescaled $\bar{t}{\bar{k}}^{\beta }$ for all *k* > k_*_ and $\bar{t}{\left(1-{\bar{k}}^{2}\right)}^{\alpha }$ for all *k* < k_*_ with the different *T*/*T*_*F*_, which give β = 1.80 (0.25) and α = −0.39 (0.16), respectively.

When quenched to unitarity, both Fermi gases before and after the interaction quench are scale invariant. Therefore, the nonequilibrium response of the gas should be universal and the evolution of the system depends only on the temperature *T*/*T*_*F*_ and the time *t*_*hold*_/τ_*F*_. After rescaling *k* with k_*_, *n*_*k*_(*t*) exhibits the universal behavior. First, we study the universal prethermal quenching dynamics of the difference of the momentum distribution $\delta \bar{n}\left(k\right)\equiv \delta n_{k}\left(t\right)/\bar{n}$, as shown in **Figure** **4**, where $\bar{n}$ is the difference of the momentum distribution of 1st QSS with the initial momentum distribution. Here, k_*_ naturally divides two different regions for the evolution. For a fixed *T*/*T*_*F*_, $\delta \bar{n}\left(k\right)$ with all momenta *k* > k* collapses into a single curve (inset Figure 4(a)), where $\bar{t}=t_{hold}/\tau_{F}$, $\bar{k}=k/k_{*}$. Actually, all $\delta \bar{n}\left(k\right)$ for the different *T*/*T*_*F*_ and all momenta *k* > k* show a universal scaling for the rescaled time and momenta of $\bar{t}{\bar{k}}^{\beta }$ where β = 1.80 (0.25) (Figure 4c). This scaling does not directly apply to the case for *k* < k_*_. Owing to the momentum distribution of ideal Fermi gases in the low temperature obeys (1 − *k* ^2^)^3/2^, we conjecture that the scaling could be (1 − *k* ^2^)^α^ for the small momenta. The data shows that $\delta \bar{n}\left(k\right)$ for all measured temperature *T*/*T*_*F*_ and momenta of *k* < k_*_ collapse onto a single curve with a rescaled time $\bar{t}{\left(1-{\bar{k}}^{2}\right)}^{\alpha }$ (Figure 4(f)), giving a universal scaling exponent α = −0.39 (0.16). These results may provide new insight into further study of momentum correlation in the quenched dynamics for Fermi gases.

**Figure 5:**
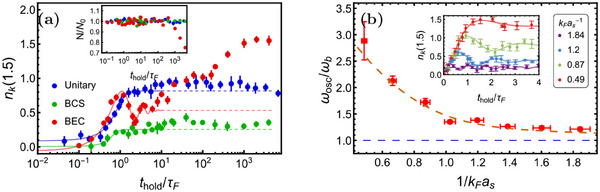
The thermalization dynamics of Fermi gases quenched to the BEC‐BCS crossover. a) The dynamical evolution of *n*_*k*_(1.5) in the BEC‐BCS crossover. Dots are the data. Blue and green solid lines are the best fits with a sigmoid function and red solid line is fitted with a sigmoid function with a sinusoidal modulation. Dashed lines are corresponding to the plateaus in the prethermalization. b) The dependence of the oscillation frequencies in the BEC region on 1/*k*_*F*_ *a*_*s*_. Red dots are data and blue dashed line denotes the asymptote of ω_*osc*_ = ω_*b*_. The orange dashed line is the prediction in bosonic contact oscillation.^[^ ^30^ ^]^ Inset is the typical early‐time dynamics for different 1/*k*_*F*_ *a*_*s*_ in the BEC side.

Next, we investigate the dynamics of the Fermi gas quenched to the BEC‐BCS crossover, as shown in **Figure** **5**. When quenched to the BCS side, the thermalization dynamics is similar to one at unitarity (green dots in Figure 5a for 1/*k*_*F*_ *a*_*s*_ = −0.64, where *a*_*s*_ is a s‐wave scattering length). Compared to the case at unitarity, *n*_*k*_ on the BCS side relaxes to a lower plateau in a shorter timescale. Although the two‐body collision rate is smaller on the BCS side, the majority of collisions at unitarity carry little energy and the transfer of energy proceeds more slowly compared to a non‐unitary regime. Therefore, the relaxation occurs more rapidly on the BCS side. The dynamics for quenched to the BEC side shows quite different evolution as Feshbach molecules with binding energy *E*_*b*_ emerge. We observe a significant oscillation in the fast growth stage, then reaching the QSS, and subsequently an increasing trend in the longer relaxation time (red dots in Figure 5a for 1/*k*_*F*_ *a*_*s*_ = 0.60).The fast growth along with the oscillation in the early‐time dynamics is determined by the atom collision and bound state scattering. We perform a curve fitting of *n*_*k*_ in the stage for the first several τ_*F*_ by a function ${\tilde{n}}_{0}{\tilde{n}}_{k}/\left({\tilde{n}}_{0}+\left({\tilde{n}}_{k}-{\tilde{n}}_{0}\right)e^{-t/\tau }\right)+\delta_{0}e^{-t/\tau_{osc}}\sin\left(2\pi \omega_{osc}t+\varphi \right)$ (Figure 5), where the first term represents the thermalization due to the collision and the second sinusoidal part denotes the contribution from the molecular bound state. When the gas is quenched close to the Feshbach resonance, the amplitudes increase while the oscillation frequencies become smaller. The measured oscillation frequencies ω_*osc*_ as a function of the measured binding energy ω_*b*_ are presented in Figure 5b. In the deep BEC region, the bound state scattering dominates the thermalization dynamics, thus the oscillation frequency approaches the binding energy. While in the strongly interacting regime 1/*k*_*F*_ *a*_*s*_ < 1, the strong collision between atoms greatly enhances the scattering between atoms and dimers, leading to a higher oscillation frequency compared to the molecular binding energy. Our measurements agree well with the predictions (dashed line).^[^ ^30^ ^]^

## 3 Conclusion

In conclusion, we have observed the dynamical evolution of Fermi gases quenched from the noninteracting to the BEC‐BCS crossover. We revealed the complex dynamics with different timescales including the prethermalization, oscillations with collective mode and bound states, very longtime thermalization and universality with the new scaling in the momentum and density distribution. Our work raises new questions for the theory for quantum thermalization in strongly interacting fermionic many‐body system, and provides an avenue to study the energy density transport,^[^ ^31^ ^]^ three‐body correlations^[^ ^32^, ^33^ ^]^ and nonequilibrium phase transition.^[^ ^34^ ^]^

## 4 Experimental Section

### Experimental Setup and Sample Preparation

Experiments were performed with a balanced mixture of ^6^Li atoms in the hyperfine states |↑〉 ≡ |*F* = 1/2, *m*_*F*_ = 1/2〉 and |↓〉 ≡ |*F* = 1/2, *m*_*F*_ = −1/2〉. The setup began with a standard magnetic optical trap (MOT) to cool atoms to a temperature of about 300 µK. A subsequent D_1_ gray molasses stage further reduced the temperature to about 80 µK with an atom number up to 3 × 10^8^. Subsequently, about 5 × 10^6^ atoms were transferred to a crossed optical dipole trap (ODT) composed of three orthogonal traps (ODT1, ODT2, ODT3 with a crossing angle of 19°) for evaporative cooling into near quantum degeneracy.^[^ ^27^ ^]^ The final cooling step involved adiabatically turning off ODT2 and switch on ODT3, realizing typically a Fermi energy $E_{F}=\hbar \bar{\omega }{\left(6N\right)}^{1/3}=k_{B}\times 1.71\mu K$ with trap frequencies ω_*z*_ = 2π × 185 Hz, ω_*x*_ = 2π × 560 Hz and ω_*y*_ = 2π × 590 Hz, where $\bar{\omega }$ is the geometric mean trap frequency and *N* ≈ 10^5^ atoms per spin state. The corresponding Fermi time τ_*F*_ = ℏ/*E*_*F*_ = 4.63µs, served as the characteristic timescale for dynamical processes.

### Initial State Preparation

Atoms were first cooled to quantum degeneracy at a magnetic field *B* = 340 G. The magnetic field was then adiabatically ramped to 528 G, where the s‐wave scattering length *a*_*s*_ between |↑〉 and |↓〉 vanishes, enabling a non‐interacting regime with negligible collisional effects.

### State Transfer and Quench

A two‐photon Raman π pulse (duration *t*_quench_ = 330 ns ≪τ_*F*_) transferred atoms from |↓〉 to the hyperfine state from |*g*_↓_〉 ≡ |*F* = 3/2, *m*_*F*_ = 1/2〉, which has a scattering length *a*_*s*_ < 10*a*_0_ for *B* > 300 G (*a*_0_ is Bohr radius). The magnetic field was then adiabatically ramped to target values between 700 and 960 G. A second Raman pulse rapidly transferred atoms back to |↓〉, quenching the system into the strongly interacting BEC‐BCS crossover regime with a holding time *t*_hold_ for dynamical evolution, during which the system evolved on timescales ranging from 0 to 10^6^τ_*F*_.

### Imaging and Momentum Distribution

After the dynamical evolution period *t*_hold_, a third Raman π pulse transferred atoms from the interacting gas back to the non‐interacting gas, eliminating collisional effects during the time‐of‐flight (TOF) expansion. Atomic density profiles were measured via absorption imaging after sufficient long TOF by an electron‐multiplying charge‐coupled device (EMCCD). For momentum‐space analysis, radial integration of density profiles *n*_*r*_(*r*) was followed by inverse Abel transformation to extract the momentum distribution *n*_*k*_(*k*). Data were averaged over 50 experimental repetitions to improve the signal‐to‐noise ratio.

### Robust Control of Interaction Quench

The two Raman beams were generated from the same high‐power fiber laser system to avoid the possible phase noise between different lasers. Several acousto‐optic modulators (AOMs) were employed to generate the frequency shifts with different Zeeman splitting. The two Raman beams are collected into one high‐power fiber with more than 120 mW for each beam. The intensity of the Raman light was stabilized by feeding back to the AOMs. The magnetic field was stabilized to 2.27 ppm using a feedback system, minimizing hyperfine level shifts (<10kHz) relative to the Rabi frequency.

## Conflict of Interest

The authors declare no conflict of interest.

## Supporting information

Supporting Information

## Data Availability

The data that support the findings of this study are available from the corresponding author upon reasonable request.
