# Supplementary material for: Quantum Thermalization Dynamics of Fermi Gases Quenched to the BEC‐BCS Crossover
Source: Adv Sci (Weinh). 2025 May 29;12(31):e07343. doi: 10.1002/advs.202507343 (PMC12376539; doi:10.1002/advs.202507343)
Supplement: Supplementary file 1 — Supporting Information [file ADVS-12-e07343-s001.pdf]

ADVANCED SCIENCE
Open Access

Supporting Information

for *Adv. Sci.*, DOI 10.1002/advs.202507343

Quantum Thermalization Dynamics of Fermi Gases Quenched to the BEC-BCS Crossover

Licheng Yi, Shuxian Yu, Meimei Wu, Shujin Deng and Haibin Wu**

Supplementary Materials for "Quantum Thermalization Dynamics of Fermi Gases Quenched to the BEC-BCS Crossover"

Licheng Yi[1], Shuxian Yu[1], Meimei Wu[1],Shujin Deng[1†], and Haibin Wu[1,2,3,4*]

[1]State Key Laboratory of Precision Spectroscopy, Institute of Quantum Science and Precision Measurement, East China Normal University, Shanghai 200062, P. R. China

[2]Shanghai Branch, Hefei National Laboratory, Shanghai 201315, China [3]Collaborative Innovation Center of Extreme Optics, Shanxi University, Taiyuan 030006, China

[4]Shanghai Research Center for Quantum Sciences, Shanghai 201315, China

[†*]To whom correspondence should be addressed: hbwu@phy.ecnu.edu.cn, sjdeng@lps.ecnu.edu.cn

S1 Methods

The experiments are performed in a balanced mixture of ^{6}Li in the two lowest hyperfine states $|\uparrow\rangle \equiv |F = 1/2, m_F = 1/2\rangle$ and $|\downarrow\rangle \equiv |F = 1/2, m_F = -1/2\rangle$. The schematic of the setup is similar to our previous work (S1–S3), as shown in Fig. S1(a). The standard magnetic-optical trap (MOT) is used to cool the atoms to about $300\,\mu K$. Then a gray molasses with the D_1 line is implemented to further cool the sample to about 80 μK with the atom number about 3×10^8. Subsequently, about 5×10^6 atoms are transferred to a crossed optical dipole trap (ODT), which consisted of ODT1 and ODT2 with an angle of about 19°. After evaporatively cooling the atoms into near quantum degeneracy, we adiabatically turn off ODT2 and turn on ODT3 to perform the final cooling. A typical absorption image with a time-of-flight (t_{tof}) expansion of the atomic cloud is employed to extract the density profiles of the atom distribution with an electron-multiplying charge-coupled device (EMCCD).

The experimental sequence to observe the dynamical thermalization evolution is shown in

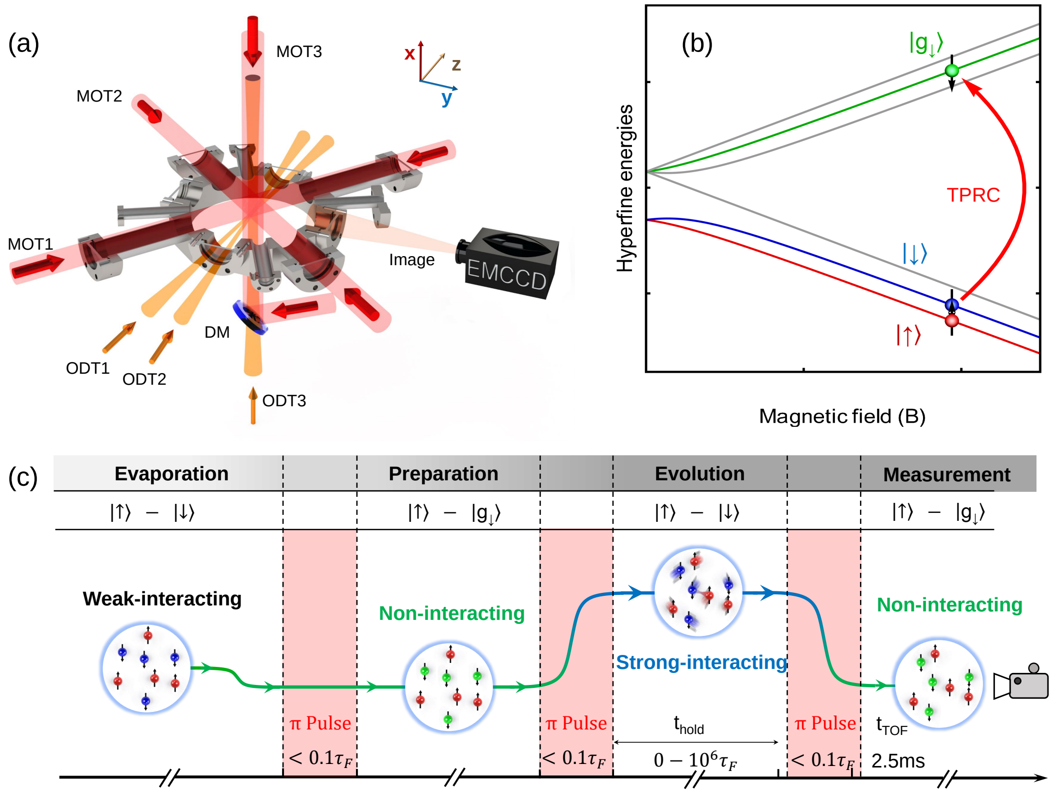

Figure S1: (a) The schematic of experimental setup. ODT (optical dipole trap), DM (dichroic mirror), EMCCD (electron-multiplying charge-coupled device), MOT (magnetic-optical trap). (b) The hyperfine energies of ground-state for ^{6}Li in a magnetic field B, for which there are six states. Here $|\uparrow\rangle \equiv |F = 1/2, m_F = 1/2\rangle$, $|\downarrow\rangle \equiv |F = 1/2, m_F = -1/2\rangle$, and $|g_\downarrow\rangle \equiv |F = 3/2, m_F = 1/2\rangle$, respectively. A two-photon Raman coupling (TPRC) of state $|\downarrow\rangle$ and $|g_\downarrow\rangle$ is used to transfer the atoms faster than a Fermi time τ_F. (c) Experimental sequence of measuring the thermalization evolution after an interaction quench.

Fig. S1(c). The atoms are first evaporatively cooled to quantum degeneracy at the weakly interacting regime of a magnetic field $B = 340$ G. Then, we adiabatically change the magnetic field B to 528 G, where a s-wave scattering length a_s vanishes for these two spin states. An optical two-photon Raman π pulse is used to completely transfer the atoms from state $|\downarrow\rangle$ to state $|g_\downarrow\rangle \equiv |F = 3/2, m_F = 1/2\rangle$ (Fig. S1(b)). The scattering length of $|g_\downarrow\rangle$ and $|\uparrow\rangle$ is predicted to be smaller than 10 a_0 (a_0 is the Bohr radius) for $B > 300$ G (S4), therefore the interaction between them is negligible. Subsequently, the magnetic field is adiabatically changed to a target value ranged from 700 G to 960 G without the loss of the atom number. Then, an optical Raman

π pulse with a duration of $t_{quench} = 330\,ns \ll \tau_F$ is employed to transfer all atoms from $|g_\downarrow\rangle$ state to $|\downarrow\rangle$ state, enabling the collisional interaction. After this ultrafast interaction quench, the system evolves with a holding time t_{hold}. Then another optical Raman π pulse is used to transfer all atoms from $|\downarrow\rangle$ state to $|g_\downarrow\rangle$ state by quenching the system from the strongly interacting to non-interacting regime again. We measure the density distribution by time-of-flight absorption images. The Rabi frequency of the two-photon Raman transition is controlled by the optical light intensity and frequency detunings, which is about $\Omega_R = 2\pi \times 1.6$ MHz and is the largest in all the energy scales in Fermi gases. Therefore, the optical Raman π pulses which transfer the atoms between $|\downarrow\rangle$ state and $|g_\downarrow\rangle$ state act as a switch of the interaction.

The completely transferring all atoms between $|\downarrow\rangle$ state and $|g_\downarrow\rangle$ state presents a technique challenge. Only 5% of the residual atoms in state $|\downarrow\rangle$ would make a strongly interacting imbalanced system (S5), causing substantial deviations in the density profile and momentum distribution from their original values. Thus the Raman laser lights, external magnetic field and energy broaden of the interacting particles are carefully manipulated to greatly suppress the decoherence for Raman transition.

We first control the coherence of the Raman laser lights. As the electronic angular momentum and the nuclear spins are decoupled in a moderate magnetic field for ^{6}Li, leading to a Zeeman splitting of $1495\,MHz$ at $B = 528$ G and $2341\,MHz$ at $B = 832$ G between $|\uparrow\rangle$ and $|\downarrow\rangle$ state, respectively. To ensure the coherence of two Raman laser lights, we employ a high power fiber laser to generate Raman beams with an acousto-optic modulators (AOM). Raman beams are injected into a polarization-maintaining (PM) optical fiber with orthogonal polarization to couple a $\sigma^+ - \pi$ Raman transition. The phase noise induced by the disturbance of the optical path and the PM fiber is measured to be smaller than $1.3\,Hz$, which is negligible compared with the two-photon Raman coupling rate. Typically total power of Raman beams is about $200\,mW$ with a diameter of about 1 mm to reduce the spatial inhomogeneity. The intensity fluctuations of the Raman beams are stabilized at the level of 10^{-4}. The frequency of the beams is red-detuned

$4\,GHz$ by the D_1 line, leading to $\Omega_R = 2\pi \times 1.6\,MHz$.

The stability of the magnetic field is another important factor to influence a realization of nearly 100% population transfer between state $|\downarrow\rangle$ and $|g_\downarrow\rangle$. The hyperfine coupling results about $3\,kHz/mG$ frequency shift at $B > 500\,G$. We develop a magnetic field locking system to realize a $2.27\,ppm$ level stability of the Feshbach magnetic field (S6), ensuring that the frequency shift from the fluctuation of external magnetic field is smaller than $10\,kHz$, which is to be more than 2 orders smaller than the Rabi frequency Ω_R of the Raman transition.

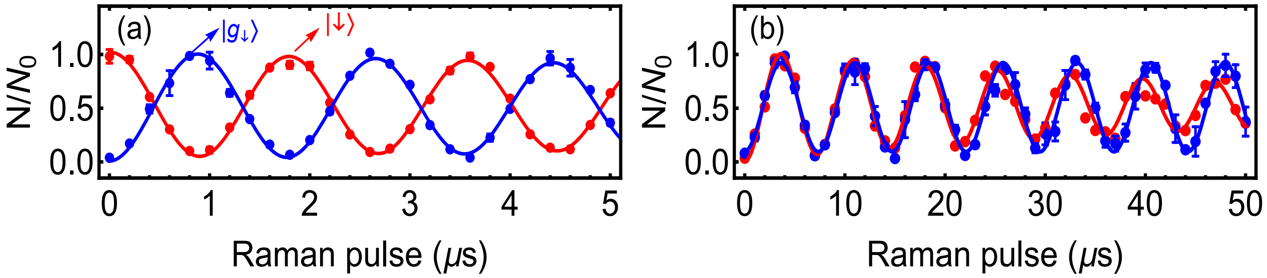

Figure S2: The Rabi oscillations between the state $|\downarrow\rangle$ and state $|g_\downarrow\rangle$. (a) Rabi oscillation for state $|\downarrow\rangle$ and state $|g_\downarrow\rangle$ when state $|\downarrow\rangle$ and state $|\uparrow\rangle$ is initially prepared at unitarity. The atom number N in state $|g_\downarrow\rangle$ (Red dots) and state $|\downarrow\rangle$ (blue dots) are normalized by their initial atom number N_0, respectively. Solid lines are the best sinusoidal fits. (b) Comparison of Rabi oscillations for the strong interaction (red dots for $B = 832\,G$, unitarity) and non-interaction (blue dots for $B = 528\,G$). There is clearly decay in the Rabi oscillation for the strong interaction. Solid lines are the best fits with a sinusoidal function of exponential decay. Each data is averaged by repeating the experiment with the same conditions for three times.

The Rabi oscillations between state $|\downarrow\rangle$ and state $|g_\downarrow\rangle$ are shown in Fig. S2(a), which is obtained by measuring the population in the states. Initially, about 10^5 atoms per spin state are prepared at unitarity in a harmonic trap with trap frequencies to be $\omega_z = 2\pi \times 33.5$ Hz and $\omega_x = \omega_y = 2\pi \times 298$ Hz, leading to the Fermi energy $E_F = \hbar\bar{\omega}(6N)^{1/3} = k_B \times 0.6\,\mu K$, where $\hbar$ is the reduced Planck constant, $\bar{\omega} = (\omega_x\omega_y\omega_z)^{1/3}$ is the geometric mean of the trap frequency, k_B is the Boltzmann constant, respectively. The corresponding Fermi time is about $\tau_F = \hbar/E_F = 12.7\,\mu s$. There is no observable loss in both states within three oscillation periods. Then the Rabi frequency is reduced to about $2\pi \times 135\,kHz$ and the measured Rabi oscillations

in both ideal and unitary regime are presented in Fig. S2(b). As is shown, the decay in the strongly interacting regime is larger than the case in the noninteracting regime, for which the large decay is caused by the atom collision in the unitarity.

Thus we demonstrate that a Raman π pulse with a duration shorter than 1 μs is fast enough to transfer nearly all atoms between the two target hyperfine states, i.e., $|\downarrow\rangle$ and $|g_\downarrow\rangle$, therefore realizing interaction quench faster than τ_F in the experiment. The residual atoms in the state $|\downarrow\rangle$ after the Raman π pulse are unobservable in the experimental resolution and we estimated that the atoms are to be less than 1%. In addition, two Raman beams propagate along the same direction, the momentum transferring for the Raman beams to atoms in the two-photon process is expected to be significantly small. To verify the validity of this powerful quench method, we measured the momentum distribution before the quench and just after the quench, as shown in Fig. S3. We first prepared a spin-balanced mixture of state $|\downarrow\rangle$ and state $|g_\downarrow\rangle$ with a Raman π pulse. Owing to the non-interaction between these states, we can accurately measure the momentum distribution. The initial density profiles were measured by a standard absorption image with a large t_{tof}. As the cloud becomes nearly isotropic, we radially integrate the density profiles and performed inverse Abel transformation to extract the momentum distribution. To get a better signal-to-noise ratio, we run the experiments 50 times with the same conditions for the average. The result in Fig. S3 shows that there is no detectable change in the momentum distribution before the quench and just after the quench, demonstrating the validity of this quench method. Owing to the initially finite atom cloud size, finite t_{tof} and resolution of the pixels of the CCD, $n_k(k)$ is not quantitatively reliable for $k < 0.3$ (gray region in the Fig. S3).

S2 The dynamical evolution of the Fermi gas quenched to the unitarity in the momentum and real space

In this section we present how we describe the quench dynamics of Fermi gases in both momentum and density space. Prethermalization emerges when the interaction strength is rapidly turned on in the non-interacting system (S7). The integrability is broken and quasi-stationary

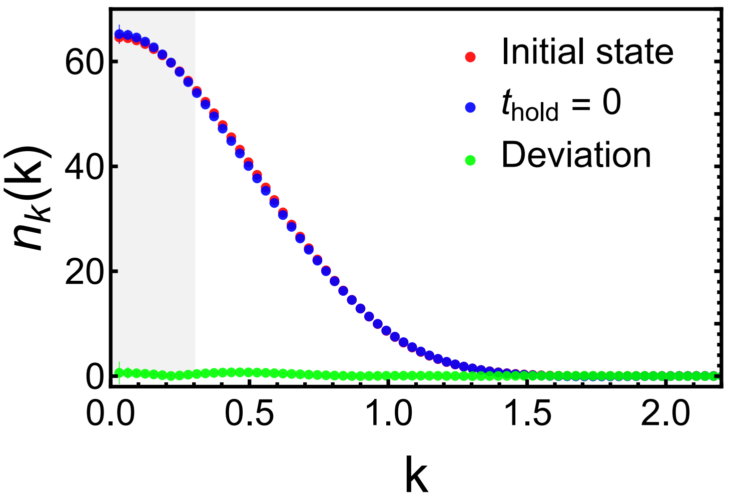

Figure S3: The momentum distribution $n_k(k)$ just before the quench (red dots) and just after the quench (blue dots). The green dots show the difference of these two momentum distributions. Gray region denotes $k < 0.3$, for which $n_k(k)$ will not be reliable due to the initial effects for the atoms.

states (QSSs) could be observed before the system truly evolves into its thermalized state. Owing to that the strongly interacting Fermi gases are the collisional stable, it provides an ideal system to explore such dynamics. Here we first observe the thermalization evolution by quenching the Fermi gas from the non-interaction to unitarity in the momentum space. We measured the time-of-flight density profiles with different t_{hold} and repeated the measurements with the same conditions for typically 30 to 60 times.

The typical quenching dynamics of n_k when quenched to unitarity in the momentum space is displayed in Fig. S4. Here, the atom number per spin is about $N = 110,000$, the Fermi time $\tau_F = 4.63\,\mu s$, $T/T_F = 0.25$. The trap frequencies of the trap are $\omega_z = 2\pi \times 185$ Hz, $\omega_x = 2\pi \times 560$ Hz and $\omega_y = 2\pi \times 590$ Hz, respectively. It clearly exhibits the complex dynamics with very different timescales. There emerge two QSSs and one final stationary state in the evolution. The whole thermalization dynamics could be divided into three stages, i.e. P_1, P_2 and P_3, corresponding to the t_{hold} of $0\sim10\tau_F$, $10\tau_F \sim 10^3\tau_F$ and $10^3\tau_F \sim 10^6\tau_F$, respectively.

The dynamics could be fitted by the following equation

$$n(k, t_{hold}) = n_0 + n_1[1 - e^{-(t_{hold}/\tau_1)^{m_1}}] + n_2[1 - e^{-(t_{hold}/\tau_2)^{m_2}}] \qquad \text{(S1)}$$
$$+ n_{osc}e^{-(t_{hold}/\tau_{osc})}\sin(2\pi\omega t_{hold} + \phi) + n_3[1 - e^{-(t_{hold}/\tau_3)^{m_3}}],$$

which consists of three sigmoid functions with different characteristic time constants τ_1, τ_2 and τ_3, and one exponentially decayed oscillation with a frequency ω and decay time constant τ_{osc}, respectively. The first term n_0 in the right side of Eq. S1 represents the initial momentum distribution. The second term $n_1[1 - e^{-(t_{hold}/\tau_1)^{m_1}}]$ represents the evolution to the first QSS. The third and fourth term describe the evolution towards the second QSS accompanied by the collective exponentially damped sinusoidal oscillation. And the last term denotes how the atoms relax to its final thermalization state.

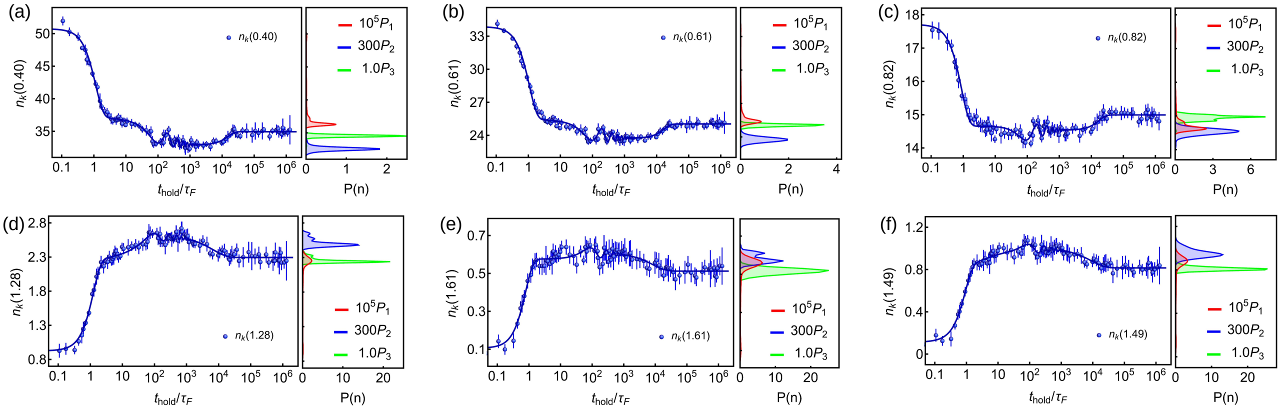

Figure S4: The dynamical evolution of the momentum distribution and its histogram for different momentum: (a) $n_k(0.40)$, (b) $n_k(0.61)$, (c) $n_k(0.82)$, (d) $n_k(1.28)$, (e) $n_k(1.49)$ and (f) $n_k(1.61)$. The evolutions of n_k are shown at every left figure, where the solid lines are for the fits from Eq. S1. The histograms for n_k are presented in the right sides. The data are interpolated with equal time intervals and then integrated to histograms. Three peaks with different scalings clearly emerge in the evolution, representing two quasi-stationary states and one steady state with very different lifetimes.

The best fit of m_1, m_2 and m_3 for $n_k(0.61)$ are given by 1.8 (0.27), 0.8 (0.14) and 2.0 (0.22), respectively, showing that the thermalization dynamics toward to first QSS is violent while the relaxation to second QSS is mildest. m_3 approaching two shows that the final thermalization

toward the stationary state is not simply exponential. Moreover, the fitted τ_1, τ_2 and τ_3 are given by $1.08\,(0.03)\,\tau_F$, $37.3\,(8.0)\,\tau_F$ and $17527\,(2744)\ \tau_F$, respectively, exhibiting three stages during the thermalization dynamics with three very different timescales. As shown in Fig. S4(b), the histogram reveals the presence of the three peaks corresponding to the 1st QSS, 2nd QSS and the final steady state in the evolution. These two QSSs have different lifetimes with two orders of magnitude difference.

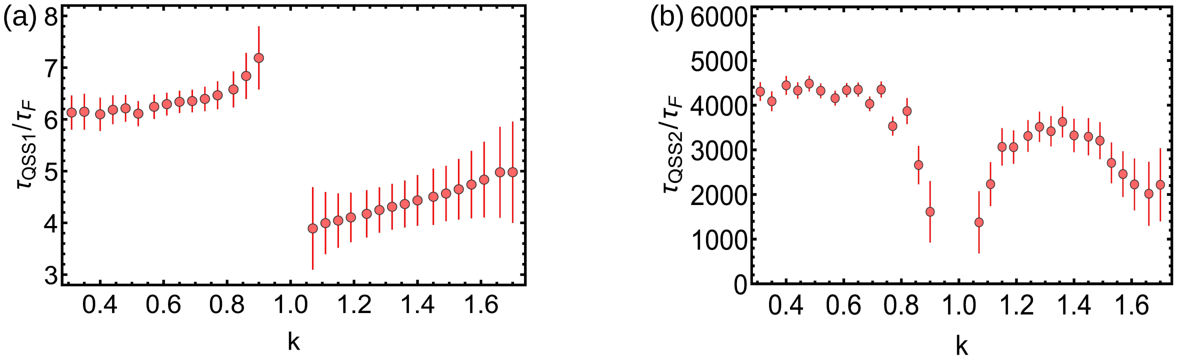

Figure S5: The measured lifetimes of two QSSs for different momentum. (a) The lifetime of the first QSS τ_{QSS1}/τ_F. (b) The lifetime for the second QSS τ_{QSS2}/τ_F. The error bars represent standard deviations.

Moreover, we further analyze the lifetime of the two QSSs, as presented in Fig. S5. The results reveal a Fano-like curve across different momentum values during the fist prethermalization, which shows two distinct regions: $k < k_*$ and $k > k_*$. Both regions exhibit an increasing trend in the lifetime with the increase of the momentum. A notable sudden drop is observed near k_*. These findings contrast with the behaviors illustrated in the following Fig. S7, where a faster thermalization process corresponds to a shorter lifetime. However, the lifetime of the second QSS exhibits a more complex and different behavior. Specifically, the lifetimes of the second QSS fast decrease as the momentum approaches k_*. We attribute this phenomenon to the intricate dynamics involved in the second thermalization process, which is closely linked to viscous hydrodynamics, thermal diffusion, and other related mechanisms. During this stage, the thermalization emerges together with collective excitations due to the hydrodynamics. Checking the second stage, the oscillated frequency for $n_k(0.61)$ is obtained by $2\pi \times 1061.2\,(31.8)$ Hz, which

is about $1.82\,(0.06)\,\omega_x$ corresponding to the radial collective mode in the unitary regime (S8,S9). The oscillation lasts about $500\,\tau_F$.

We also investigate the dynamical evolution of the population after the interaction quench. The population for each momentum shell is expressed as $4\pi\delta n(k)k^2$, where $\delta n(k) = n_k(k, t_{hold}) - n_k(k, 0)$, as shown in Fig. S6. The population for each shell barely changed for $t_{hold} < 0.1\ \tau_F$. After these holdtimes, the population kept decreasing for lower momenta while increasing for larger one, indicating the population transferring from the low momenta to the high momenta during the evolution. There are some special momenta where the population didn't change, i.e. $\delta n(k) = 0$, as time evolves. After the quench, this non-evolution momentum tends to gradually decrease and approach a fixed value after first prethermalization stage within the first several τ_F (yellow dots in Fig. S6). We define this special momentum as k_*, which is about 0.97 at $T/T_F = 0.25$.

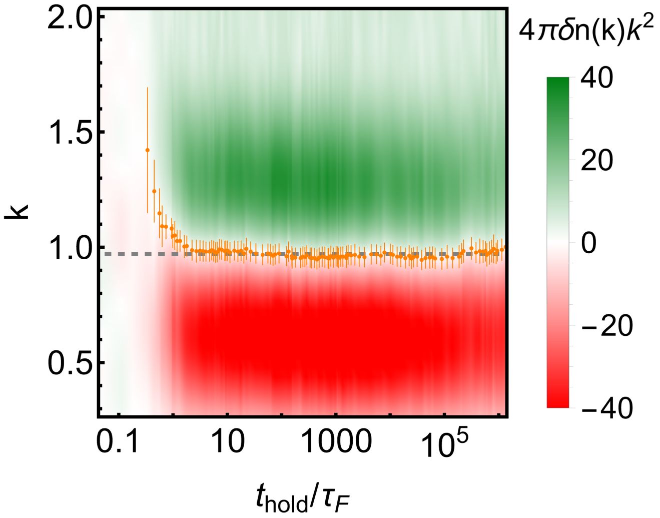

Figure S6: The dynamical evolution of atom population $4\pi\delta n(k)k^2$ for different momentum shells. Yellow dots denote for the momenta where $\delta n(k)$ is zero. These special momenta devides two parts for the evolution, with a decrease in the population smaller than this special momentum and an increase in the population larger than this special momentum. Gray line is a best fit to give a crossover momentum k_*.

The quench dynamics is momentum dependent. The momentum distribution $n_k(k)$ has different evolution for $k < k_*$ and $k > k_*$. In the first stage, $n_k(k)$ experiences a fast decreasing

for $k < k_*$ and an increasing for $k > k_*$. After these relaxations, $n_k(k)$ evolves into a plateau, the prethermalized state. The time constants are extracted by Eq. S1, as shown in Fig. S7. τ_1 exhibits a divergent behavior when momentum approached k_* (a dashed line in Fig. S7). $n_k(k)$ reaches its quasi-stationary stage faster for higher momenta. The slowest dynamics emerges when the momenta reaches k_* from the low momentum side while $n_k(k)$ has the most violently relaxation for the momentum just larger than k_*. τ_2 and τ_3 have a similar momentum dependence as τ_1, but with very large values. Typically, τ_2 and τ_3 are about two and four orders of the magnitude of τ_1.

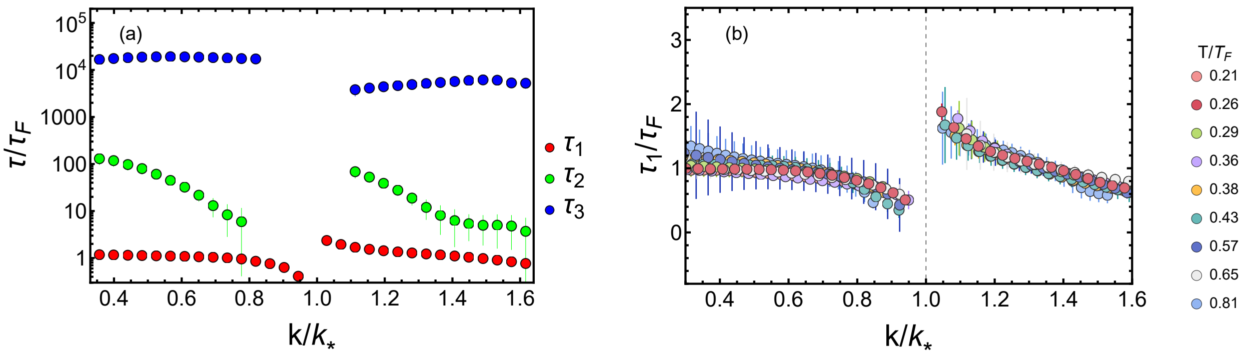

Figure S7: τ/τ_F as a function of the scaled momentum k/k_* for different evolution stages (a) and its universality (b) for different dimensionless initial temperature T/T_F for the first prethermal dynamics. It shows the dispersion-like behavior and is divergent near k_*.

We observe the quenching dynamics for different dimensionless initial temperature T/T_F, which shows a similar evolution. τ_1 as a function of k/k_* and t_{hold}/τ_F for T/T_F ranged from 0.21 to 0.81 is displayed in Fig. S7. For the higher T/T_F, k_* becomes large and is determined by the thermal wavelength. Owing to that both gases are scale invariance before and after the quench, τ_1/τ_F shows a universality with all data collapsing on one curve for the different temperatures after the momentum rescaling with k_*, as is shown in Fig. S7.

We also observe the quench dynamics in the real space by measuring the cloud size after the interaction quench. Being different from measuring its momentum distribution $n_k(k)$, we directly measured the atomic cloud after the interaction quench with the second Raman π pulse to transfer the atoms into state $|g_\downarrow\rangle$. The atom density distributions are directly measured with

$1\,ms$ to $2\,ms$ t_{tof} expansion in the strong interaction after the quench. The column density along the axial or radial direction is fitted by a simple Gaussian function as $A_0 + A_1 e^{-x_i^2/\sigma_i^2}$ to get the cloud sizes σ_z and σ_r.

Figure S8 shows a typical dynamics of the axial cloud size after quenching into the unitarity. The evolution of the atomic cloud size can be described by

$$\sigma_i/\sigma_{i0} = a_0 + a_2[1 - e^{-(t_{hold}/\tau_2)^{m_2}}] + a_{osc} e^{-(t_{hold}/\tau_{osc})} \sin(2\pi\omega_{osc} t_{hold} + \phi) + a_3[1 - e^{-(t_{hold}/\tau_3)^{m_3}}], \quad \text{(S2)}$$

where σ_i $(i = r, z)$ is the radial or axial mean cloud size, $\sigma_{i0}(i = r, z)$ is the initial mean cloud size.

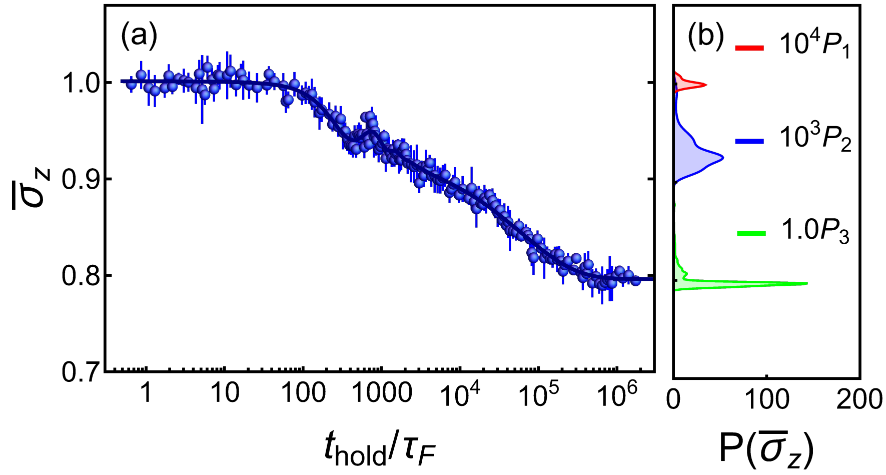

Figure S8: The dynamical evolution of the axial mean cloud size ($\bar{\sigma}_z = \sigma_z/\sigma_{z0}$) (a) and its histogram (b). The axial cloud size keeps unchanged in $0 \sim 100\tau_F$ and then begins to relax with the oscillation into the QSS at $10^3\tau_F$. After the system is trapped in this QSS for several thousands of τ_F, the cloud size very slowly evolves into the final state. Three peaks emerge in the histogram, representing the unchanged cloud size, one QSS and one stationary state, respectively.

The thermalization dynamics of atomic cloud size is different from its momentum evolution. There is no change for the cloud size during the first 100 τ_F, while $n_k(k)$ violently relaxes to its first QSS in this stage. After this stage, the cloud size begins to thermalize and oscillates into the QSS at about $10^3\tau_F$. After remaining on this state for several thousands of τ_F, the cloud size slowly relaxes to its final stationary state. The histogram (Fig. S8(b)) shows three

peaks corresponding to an unchanged stage of the cloud size, one QSS and one stationary state, respectively. The best fit with Eq. S2 to the radial cloud size gives that the oscillation frequency ω_{osc} is $2\pi \times 977.8$ Hz, which is about $1.75\,\omega_x$, close to the oscillation frequency measured in the momentum space, revealing collective radial mode emerged in both kinetic and potential energy space. The corresponding parameters of m_2 and m_3 are 0.99 (0.38) and 0.62 (0.09), respectively. However, the value of τ_2 is found to be far larger than its value in momentum evolution, up to $1213\,(285)\,\tau_F$. The thermalization towards the final quasi-stationary state evolves with a time constant τ_3 of about $60075\,(9982)\,\tau_F$.

S3 Relaxation dynamics of atomic momentum distribution when quenched to the BEC-BCS crossover

When quenched into the BEC-BCS crossover, the dynamics shows very different evolution. As mentioned in the maintext, we find the low plateau and the rapid relaxation in the 1st QSS when quenched into the BCS side. We investigate the amplitudes $|\Delta n_k| = |n_0 - n_1|$ and the characteristic time constants τ_1 for different momentums after quenched into the unitarity and BCS side, as shown in Fig. S9. Here n_0 and n_1 are obtained from the fit to data with the Eq. S1. When comparing the quenching dynamics between the unitary and BCS regime, we do find the lower plateau in the BCS side and rapid relaxation in the first QSS. The collisional rate is related to the cross section which has unitary-limited maximum of $4\pi/k^2$ at the Feshbach resonance, which means that it is large for pairs of particles with small relative momenta. Therefore, the collisions carry relatively small amounts of energy. The Fermi gas experiences a lower collisional rate in the BCS side owing to the small scattering length. It has been investigated that the number of collisions required to thermalize away from unitarity is 3∼4 times less than the case for the unitary gas by using Monte-Carlo methods (S10, S11). Since at unitarity the majority of collisions carry little energy, the transfer of energy proceeds more slowly compared to a non-unitary regime with the same collision rate. This could slow down thermalization at unitarity.

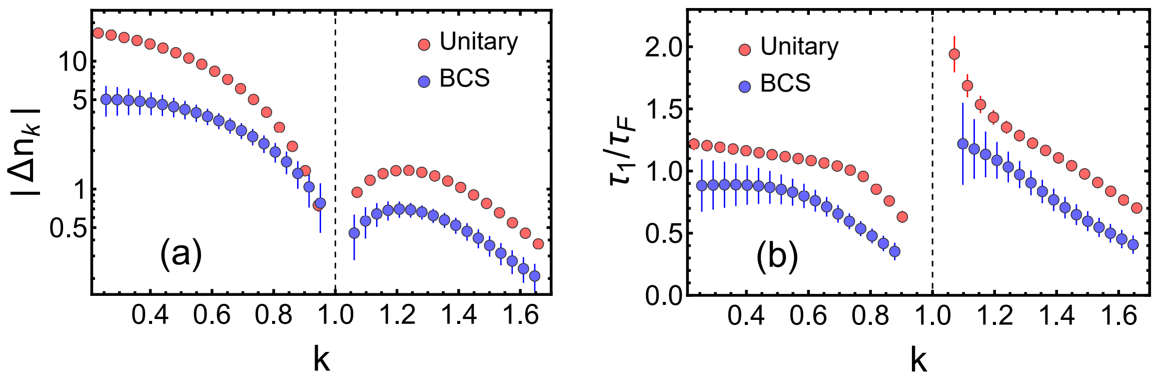

Figure S9: Δn_k (a) and τ_1/τ_F (b) as a function of the momentum k for the unitary (red dots) and BCS regime (blue dots) for the first QSS.

References

S1. S. Deng, Z.-Y. Shi, P. Diao, Q. Yu, H. Zhai, R. Qi, and H. Wu, Science 353, 371 (2016).

S2. S. Deng, P. Diao, Q. Yu, A. del Campo, and H. Wu, Phys. Rev. A 97, 013628 (2018).

S3. S. Deng, A. Chenu, P. Diao, F. Li, S. Yu, I. Coulamy, A. Campo, and H. Wu, Sci. Adv. 4, eaar5909 (2018).

S4. M. Bartenstein, A. Altmeyer, S. Riedl, R. Geursen, S. Jochim, C. Chin, J. Hecker Denschlag, R. Grimm, A. Simoni, E. Tiesinga, C. J. Williams, and P. S. Julienne, Phys. Rev. Lett. 94, 103201 (2005).

S5. B. Mukherjee, Z. Yan, P. B. Patel, Z. Hadzibabic, T. Yefsah, J. Struck, and M. W. Zwierlein, Phys. Rev. Lett. 118, 123401 (2017).

S6. X. Liu, Y. Rui, L. Zhang, Y. Wu, and H. Wu, Acta Phys. Sin. 71, 145205 (2022).

S7. K. Mallayya, M. Rigol, and W. De Roeck, Phys. Rev. X 9, 021027 (2019).

S8. J. Kinast, A. Turlapov, and J. E. Thomas, Phys. Rev. Lett. 94, 170404 (2005).

S9. A. Altmeyer, S. Riedl, C. Kohstall, M. J. Wright, R. Geursen, M. Bartenstein, C. Chin, J. H. Denschlag, and R. Grimm, Phys. Rev. Lett. 98, 040401 (2007).

S10. C. R. Monroe, E. A. Cornell, C. A. Sackett, C. J. Myatt, and C. E. Wieman, Phys. Rev. Lett. 70, 414 (1993).

S11. G. M. Kavoulakis, C. J. Pethick, and H. Smith, Phys. Rev. Lett. 81, 4036 (1998).
